# Bimetallic Ni–Ru and Ni–Re Catalysts for Dry Reforming of Methane: Understanding the Synergies of the Selected Promoters

**DOI:** 10.3389/fchem.2021.694976

**Published:** 2021-07-07

**Authors:** Andrea Álvarez Moreno, Tomás Ramirez-Reina, Svetlana Ivanova, Anne-Cécile Roger, Miguel Ángel Centeno, José Antonio Odriozola

**Affiliations:** ^1^ Estado Sólido y Catálisis Ambiental, Departamento de Química, Facultad de Ciencias, Universidad Nacional de Colombia, Ciudad Universitaria, Bogotá, Colombia; ^2^ Centro Mixto Universidad de Sevilla-CSIC, Instituto de Ciencia de Materiales de Sevilla, Sevilla, Spain; ^3^ Department of Chemical and Process Engineering, University of Surrey, Guildford, United Kingdom; ^4^ ICPEES, équipe Energie et Carburants pour un Environnement Durable, UMR CNRS, Strasbourg, France

**Keywords:** dry reforming, Ni-based catalyst, ruthenium, rhenium, deactivation, coke

## Abstract

Designing an economically viable catalyst that maintains high catalytic activity and stability is the key to unlock dry reforming of methane (DRM) as a primary strategy for biogas valorization. Ni/Al_2_O_3_ catalysts have been widely used for this purpose; however, several modifications have been reported in the last years in order to prevent coke deposition and deactivation of the samples. Modification of the acidity of the support and the addition of noble metal promoters are between the most reported strategies. Nevertheless, in the task of designing an active and stable catalyst for DRM, the selection of an appropriate noble metal promoter is turning more challenging owing to the lack of homogeneity of the different studies. Therefore, this research aims to compare Ru (0.50 and 2.0%) and Re (0.50 and 2.0%) as noble metal promoters for a Ni/MgAl_2_O_4_ catalyst under the same synthesis and reaction conditions. Catalysts were characterized by XRF, BET, XRD, TPR, hydrogen chemisorption (H_2_-TPD), and dry reforming reaction tests. Results show that both promoters increase Ni reducibility and dispersion. However, Ru seems a better promoter for DRM since 0.50% of Ru increases the catalytic activity in 10% and leads to less coke deposition.

## Introduction

The concepts of “circular economy” and “CO_2_ utilization” are among the most promising strategies in order to deal with global warming and energy storage. The ideal scheme that could couple both concepts lays in the carbon capture and utilization (CCU) approach where waste CO_2_ can be captured and utilized as feedstock in later reactions.

One of the most common CO_2_-rich waste streams is biogas. Biogas is a gas mixture generated by the anaerobic digestion of organic matter (Sarkar et al., 2018; Ferreira et al., 2019). Although its composition is deeply dependent of the digestion process, it is dominated by CO_2_ and CH_4_. Valorization of this waste stream can be carried out though the dry reforming process (DRM) (Aramouni et al., 2018; Aziz et al., 2019).

DRM (Eq. 1) is a highly endothermic reaction in which CH_4_ and CO_2_ are converted to syngas (H_2_ + CO), which is a vital feedstock in generating other useful chemicals such as methanol, olefins, and ammonia (Aziz et al., 2019).

$$CH_{4}+CO_{2}\to 2CO+2H_{2}\Delta_{r}H_{298}^{\circ }=247kJ/mol.$$ (1)

This reaction has been proposed as one of the most promising technologies for utilization of these two greenhouse gases (Wang et al., 1996; Aramouni et al., 2018; Aziz et al., 2019). However, an industrial approach of this process has not been established owing to the low activity and coke formation on the catalysts during the prolonged reaction times.

Catalysts for DRM are based on a highly dispersed active metal over a metal oxide support. Between the reported active metals, Ni stands out owing to its availability, low cost, and remarkable catalytic activity. Nevertheless, it is widely reported that Ni-based catalysts are prone to deactivation by particle sintering and coke deposition (Bradford and Vannice, 1999; Guczi and Erdohelyi, 2012; Ryi et al., 2014). Fortunately, stability of Ni-based catalysts can be improved by the addition of promoters and by the modification of the acidity of the support.

Several studies propose that the addition of basic elements could indeed change the support acidity. Wang et al. (Wang and Lu, 2000) reported that the addition of basic elements as Na or Mg reduces the carbon formation in a 13.4% wt. Bobadilla et al. (Penkova et al., 2011; Bobadilla et al., 2014) established that 10% of MgO allowed the modification of the support acidity and improved the Ni dispersion. In the same line, Alipour et al. (2014) reported that the addition of MgO reduced the coke formation on a Ni/Al_2_O_3_ catalyst and even improved its catalytic activity. Regarding the promoters, it is widely accepted that small additions of noble metals (∼1–5 %wt) improve the stability and activity of nickel-based catalysts (Wang et al., 1996; Abdel Karim Aramouni et al., 2017; Mohd Arif et al., 2019). Ruthenium and rhenium are between the most interesting and accessible promoters. Rhenium is a widely known promoter in the Pt-based catalysts for reforming reactions. Several studies indicate that Re reduces the sintering of Pt particles (Azzam et al., 2007; 2008), and it is widely reported that its presence improves selectivity and activity (Richardson et al., 2001; King et al., 2010; Carvalho et al., 2012). Owing to these facts, Re has been proposed to be a good promoter in Ni-based catalysts. Few studies have been developed within the Re–Ni system; however, data suggest that Re addition could increase the chemisorbed H_2_, boost the Ni dispersion, and decreas the coke deposition on the catalyst’s surface (Borowiecki et al., 2008; Daorattanachai et al., 2018; Wang, 2020; Xu et al., 2020). Ruthenium, on the other side, has been widely used at important industrial processes including hydrogenation, Fischer–Tropsch synthesis, ammonia synthesis, and steam reforming (Baranowska and Okal, 2016; Zhu et al., 2020). It has been reported that Ru increases the stability and activity of the Ni/MgAl_2_O_4_ catalyst in reforming reactions, but it is extremely sensitive to the synthesis method (Crisafulli et al., 2002; Álvarez M et al., 2015). Zhou et al. (2018) reported that Ru increased the activation barrier for the CH_4_ disproportionation slowing carbon deposition rate and accelerated carbon gasification by CO_2_. Wysocka et al. (2019) reported that the addition of Ru to Ni/MgAl_2_O_4_ catalysts enhanced the methane conversion and shifted the H_2_/CO ratio to lower values. Thereby, in line with the previous comments, this study compares the effect of Re and Ru as promoters on a Ni/MgAl_2_O_4_ catalyst, in terms of its different physicochemical properties, stability, and ability of resistance to deactivation.

## Materials and Methods

### Catalyst Preparation

Support modification was prepared by wet impregnation of a commercial high-purity γ-alumina (Sasol) by an ethanolic solution of Mg(NO_3_)_2_•6H_2_O (Aldrich) in order to obtain 10% wt of MgO. The full procedure is described elsewhere (Álvarez M et al., 2015). Active phase incorporation was achieved by wet impregnation of the metal precursors: Ni(NO_3_)_2_.6H_2_O (Panreac), Ru(NO) (NO_3_)_3_ (Johnson Matthey), and NH_4_ReO_4_ (Aldrich).

Monometallic Ni catalysts (Ni sample) were prepared by adding an ethanolic solution of Ni(NO_3_)_2_.6H_2_O to the modified support in order to achieve a load of 15% wt. Catalyst is then dried and calcined at 500°C for 3 h.

For the bimetallic ReNi and RuNi catalyst (2RuNi, 0.5RuNi, 2ReNi, and 0.5ReNi), a mixed solution of dissolved Ni(NO_3_)_2_.6H_2_O+ NH_4_ReO_4_ or Ni(NO_3_)_2_.6H_2_O+ Ru(NO) (NO_3_)_3_ was added to the modified support in order to achieve Ni loadings of 15% wt and Re or Ru load of 2.0 and/or 0.50% wt. After impregnation, all catalysts were dried at 120°C overnight and then calcined in air at 500°C for 5 h.

### Catalysts Characterization

The chemical composition of the samples was determined by X-ray fluorescence spectrometry (XRF) in a PAnalytical AXIOS PW440 sequential spectrophotometer with a rhodium tube as source of radiation.

The textural properties were studied by N_2_ adsorption measurements at liquid nitrogen temperature. The experiences were carried out by means of a Micromeritics ASAP 2010 equipment. Before analysis, the samples were degassed for 2 h at 250°C in vacuum.

X-ray diffraction (XRD) analysis was performed on an X’Pert Pro PANalytical Diffractometer. Diffraction patterns were recorded with Cu K radiation (40 mA, 45 kV) over a 2θ-range of 10–80° and a position-sensitive detector using a step size of 0.05° and a step time of 1.0 s. Crystallite size calculations were performed based on the Scherrer equation (Neimark et al., 2008):

$$d=\frac{K\;\lambda }{\beta \;cos\theta }$$ (2)

where *d* is the crystallite size in nm, K is a constant (shape factor = 0.94), λ is the wavelength of the X-ray radiation employed (λ = 0.154 nm), β is the full width at half maximum (FWHM) expressed in radians, and θ is the angular position of the peak maximum.

The temperature-programmed reduction (H_2_-TPR analysis) was carried out in a Micromeritics AutoChem II 2920 equipment with a TCD detector. The analysis was performed with 100 mg of fresh catalyst under 25 ml min^−1^ of a 10% H_2_/Ar mixture. The temperature was increased from room temperature to 950°C with a rate of 10°min^−1^. Reducibility calculations were performed with the relationship between the theoretical H_2_ moles consumed by all metal species (assuming NiO, RuO_2_, and ReO_2_ and taking into account the metal content evidenced by XRF analysis) and the real H_2_ consumption by the sample.

H_2_-TPD experiments were also carried out in a Micromeritics AutoChem II 2920 equipment. However, in this case, the analysis was performed with 200 mg of fresh catalyst under 50 ml min^−1^ of a mixture 10% H_2_/Ar. The temperature was increased from room temperature to 850°C with a rate of 10°Cmin^−1^. The final temperature was maintained for 3 h in order to simulate the pretreatment protocol of the samples before the reaction. After reduction, a flow of 25 ml min^−1^ of Ar was passed through the sample as the temperature is reduced until 50°C. Afterward, 30 pulses of H_2_ are sent to the sample in order to assure complete saturation of the surface. Later, H_2_ physisorbed is cleaned from the surface during 1 h with a flow of 25 ml min^−1^ of Ar. Finally, the temperature is raised at 10°Cmin^−1^ until 950°C in order to desorb the chemisorbed H_2_.

Temperature programmed oxidation (TPO) was used to quantify the carbonaceous deposits on the catalysts after 50 h of reaction. The temperature was ramped at a rate of 15 °Cmin^−1^ from room temperature to 900°C, while a 1% mixture of oxygen in helium was passed through the 0.020 g of the catalyst at 1 atm. Quantification of carbon deposition was possible by monitoring the oxidation gases by online mass spectrometry in a PFEIFFER vacuum equipment.

### Catalytic Activity

The dry reforming of methane was carried out in a fixed bed quartz reactor. Prior to the reaction, the catalyst was reduced in 50% H_2_/N_2_ at 850°C for 3 h (100 Nml min^−1^). After the pretreatment, a feed mixture of CH_4_/CO_2_/N_2_ 35/35/30 was introduced into the reactor. The total space velocity was equal to 110 N L h^−1^ g^−1^. The reforming tests were performed at 750°C during 6 and 50 h to evaluate differences in catalytic activity and stability. The effluent gases were analyzed by using a Micro Gas Chromatograph (Agilent) equipped with two columns: Poraplot U and Molecular Sieve 5 A.

## Results and Discussion

### Catalyst Characterization

Chemical composition of the samples, confirmed by XRF (Table 1), showed general agreement between the theoretical and experimental compositions. All bimetallic samples present Ni content around the expected value (∼15%). However, slight differences were evidenced in the amount of promoter. In the Re–Ni samples, the amount of Re was found to be ∼0.3% higher than expected, unlike what is evidenced in the Ru–Ni samples, where the amount of promoter is around ∼0.3% lower. Although differences in the noble metal content may seem significant, the variation is in agreement with the uncertainty of the measurement, where variations in 0.5 to 1% are reported (Richard and Rousseau, 2001).

**TABLE 1:** Textural properties and composition of the synthesized samples.

Sample	Ni% wt	Re% wt	Ru% wt	S_BET_ m^2^/g	Pore volume cm^3^/g	Pore size nm
Modified Support	—	—	—	123	0.37	9.4
Ni	17.2	—	—	104	0.30	9.1
0.5ReNi	15.3	0.81	—	99	0.28	8.7
2ReNi	14.0	2.33	—	100	0.28	8.7
0.5RuNi	15.2	—	0.42	102	0.28	8.6
2RuNi	15.1	—	1.61	98	0.27	8.6

The monometallic sample presents a slight higher content of Ni than expected, and the modified support successfully incorporated 9.8% of MgO [support XRF characterization shown elsewhere (Álvarez M et al., 2015)].

**FIGURE 1:**
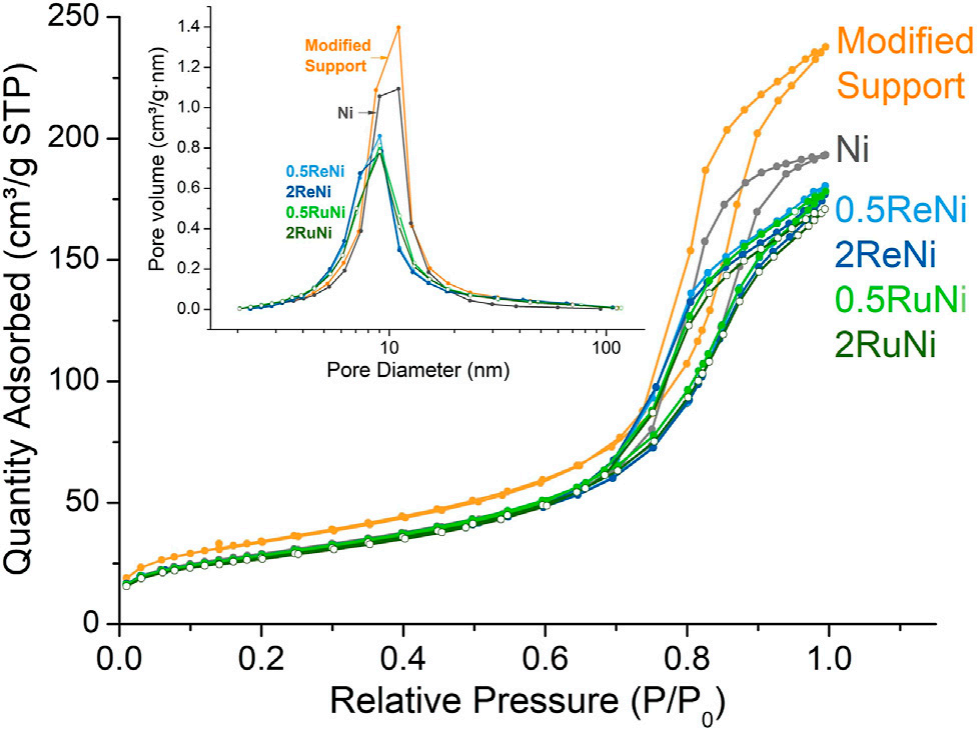
Adsorption–desorption isotherms and pore size distribution of the synthesized samples.

Regarding the textural properties, adsorption and desorption isotherms are of type IV (Figure 1) which indicates the mesoporous nature of all the samples (Leofanti et al., 1998; Thommes et al., 2015). The values of surface area, pore volume, and pore size of the modified support agree with the reported values of a modified Al_2_O_3_ (Profeti et al., 2009; Bobadilla et al., 2015). However, it is clearly observed that these values decrease with the metal impregnation regardless of the noble metal used, implying some porosity blockage owing to the synthesis process.

**FIGURE 2:**
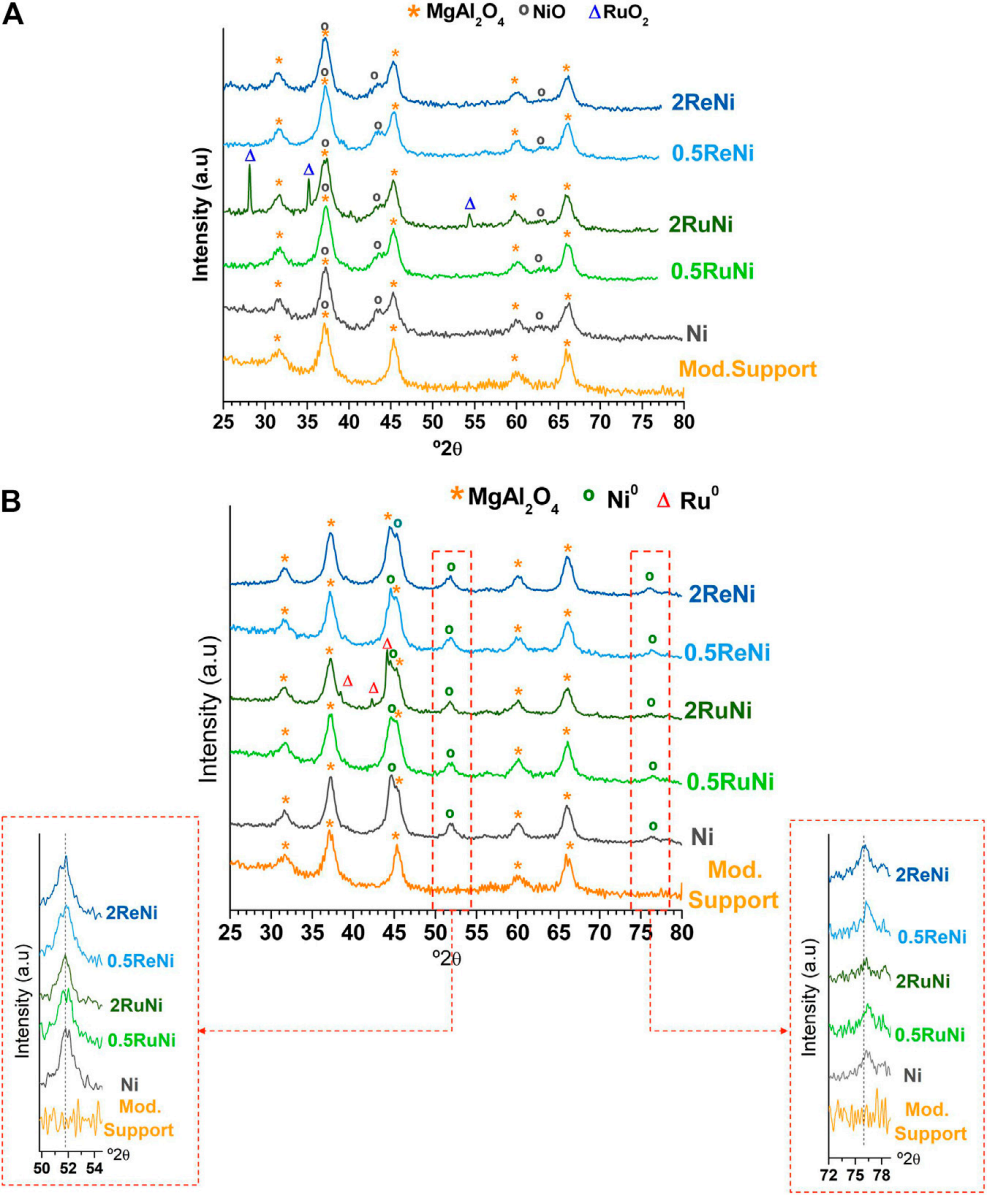
XRD patterns: **(A)** calcined samples and **(B)** reduced samples.

Figure 2 displays the XRD patterns of the calcined (Figure 2A) and reduced (Figure 2B) samples. In both cases, the diffractograms are dominated by the modified support pattern with peaks at 31.5, 37, 45, 59, and 66° 2θ corresponding to the MgAl_2_O_4_ spinel (JCPDS 00-021-1152) (Andraos et al., 2019; Wysocka et al., 2019). The presence of NiO was confirmed in all the calcined samples by the diffraction peaks at 43.3 and 63.7° 2θ (JCPDS 44-1159) (Profeti et al., 2009; Andraos et al., 2019). A slight broadening of the peak at 43.3° 2θ suggests NiO crystallite size is reduced when the impregnation is performed along a second noble metal; however, no clear conclusions can be withdrawn regarding the NiO crystallite size owing to the overlapping with the peak at 45° 2θ of the MgAl_2_O_4_ spinel.

The sharp peaks at 28.1, 35.1, and 54.4° 2θ observed in the sample with 2% of Ru (2RuNi) indicate the presence of large RuO_2_ particles (JCPDS 40-1290) (Baranowska et al., 2014; Mahfouz et al., 2020). The crystallite size of RuO_2_ in this sample was found to be around 35 nm by Scherrer calculations. On the other hand, no RuO_2_ peaks are evidenced in the sample with 0.5% of Ru (0.5RuNi), implying a highly dispersed RuO_2_. No rhenium oxides are evidenced in the 2ReNi and 0.5ReNi samples, indicating a highly dispersed and/or amorphous oxide. Similar results have been reported in the catalyst based in Re over Al_2_O_3_, where no evidence of rhenium oxides is observed (Claridge et al., 1994; Okal et al., 1999; Baranowska et al., 2014; Baranowska and Okal, 2016; Daorattanachai et al., 2018).

After reduction (Figure 2B), Ni is evidenced as Ni^0^ by the reflections at 44.6, 51.9, and 76.5° 2θ assigned to Fm3m Ni phases with the lattice constant of 3.523 A˚ (JCPDS 87-0712) (Cai et al., 2014). After the Scherrer analysis of the peak at 51.9° 2θ, it was shown that the average crystal particle size of Ni^0^ was around 6.7 nm in the monometallic sample and in the ReNi samples (Table 2); however, a slight increase in the Ni^0^ crystallite size was evidenced when Ru was used as a promoter.

**TABLE 2:** Crystallite size of metal oxides and reduced metals.

Sample	Ni^0^ (nm)	Ru^0^ (nm)	Re^0^ (nm)
Ni	6.7	—	—
0.5ReNi	6.6	—	^a^
2ReNi	6.6	—	^a^
0.5RuNi	7.1	^a^	—
2RuNi	8.1	38.3	—

a Not Evidenced.

Regarding the latter, large Ru^0^ crystals (∼38 nm) are observed in the 2RuNi sample which agrees with the large RuO_2_ crystals observed in the calcined sample. No Ru^0^ peaks are observed in the 0.5RuNi sample, and neither Re^0^ diffraction peaks are observed in the 2ReNi or 0.5ReNi samples denoting a high dispersion of both metals after reduction. The existence of a RuNi or ReNi alloy was analyzed by the shifting of the Ni^0^ peak at 51.9 and 76.5° 2θ (Figure 2B inset). Although some authors report a clear shift denoting an alloy (Wang et al., 2006; Zhou et al., 2018; Wang, 2020; Xu et al., 2020), no shifting was observed in the present samples.

➢ Crystallite size calculation of Ni^0^ and Ru^0^ was analyzed by the peaks at 51.8 and 42.2° 2θ, respectively.

**FIGURE 3:**
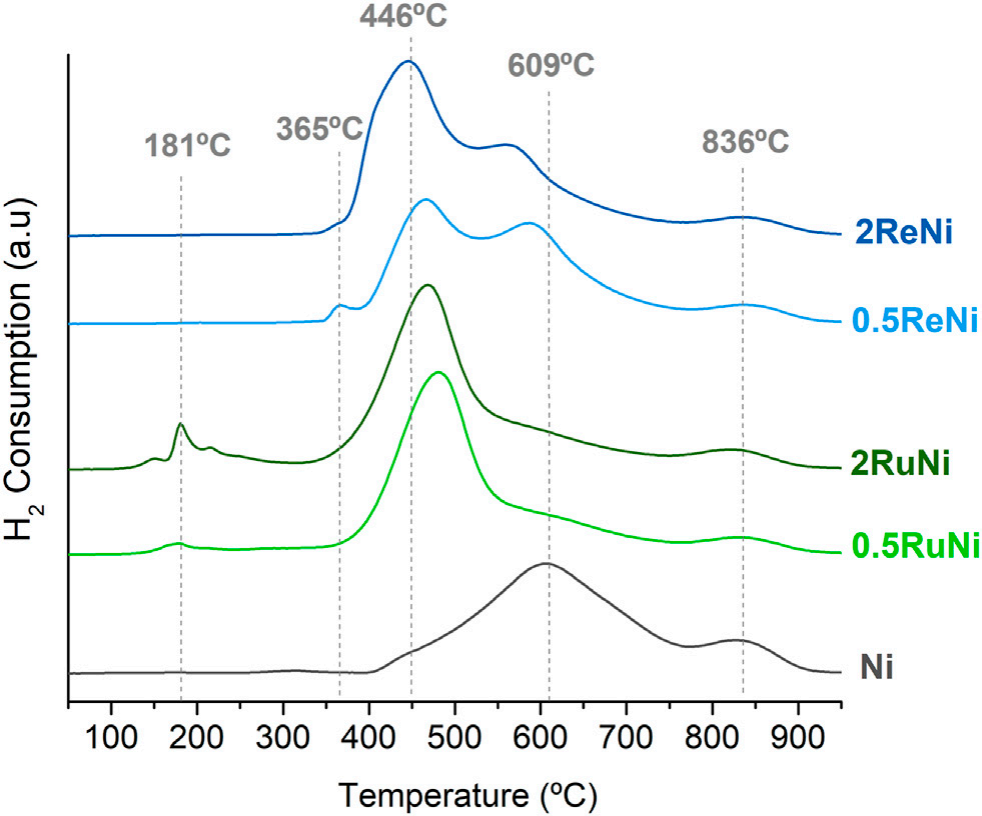
H_2_-TPR profiles of the synthesized samples.

The TPR was performed in order to evaluate the interaction of Ni with the promoters and the support (Figure 3). The reduction pattern of the monometallic sample shows two clear regions, one broad reduction peak around 600°C and a second one at 836°C. According to published studies (Profeti et al., 2009; Yaakob et al., 2013; Andraos et al., 2019), NiO species are reported to be reduced in a range from 400 to 600°C, depending on their interaction with the support. The broad peak centered at 609°C of the monometallic sample indicates that the well-dispersed NiO particles evidenced in XRD had a medium–strong interaction with the support. However, a strong metal-support interaction is evidenced in the peak at 836°C that implies the insertion of Ni in the support structure forming the NiAl_2_O_4_ spinel (Alipour et al., 2014; Luisetto et al., 2017). This latter structure was not identified in the XRD analysis since its XRD pattern overlaps with the one of the MgAl_2_O_4_ spinel.

Regarding the samples with Ru, three regions are identified. The first reduction peaks observed around 180°C can be attributed to the reduction of RuO_2_ species (Andraos et al., 2019). It has been reported that the presence of different peaks in this region can be ascribed to the different interactions with the support or different RuO_2_ crystallite size particles; peaks around 150°C denote small RuO_2_ species with a weak interaction with the support, whereas peaks near 200°C can be ascribed to large RuO_2_ particles with a higher support interaction (Mahfouz et al., 2020). As observed, the 2RuNi sample displays reduction peaks up to 210°C, which agrees with the large RuO_2_ crystals observed in XRD, whereas the sample with 0.5% of Ru presents a small reduction process near 160°C which agrees with a highly dispersed RuO_2_. The second reduction peak observed in these samples starts around 360°C up to 750°C. This wide peak represents the reduction of NiO species in interaction with Ru. The addition of Ru lowers the reduction temperature of the NiO species. This effect has been widely reported as the spillover effect, where the active H_2_ dissociates from the reduced Ru, migrates to the NiO species, and facilitates its reduction process (Mohd Arif et al., 2019). Likewise, the increased reducibility of the sample, calculated by its H_2_ intake (Table 3), supports this statement. Hence, the evidenced shifting, and the increased reducibility, denotes a remarkable interaction between the metals (Crisafulli et al., 1999). The last reduction peak observed in these samples corresponds to the reduction of Ni in the NiAl_2_O_4_ spinel; however, it is evidenced that the intensity of this peak is lower in the promoted samples with Ru and Re, compared with the intensity in the monometallic sample, implying that the addition of these two promoters somehow prevents the formation of the NiAl_2_O_4_ structure (Andraos et al., 2019).

**TABLE 3:** Reducibility and data extracted from the H_2_-TPD.

Sample	Reducibility (%)	Chemisorbed H_2_ (mL/g)	Dispersion (%)	Metallic surface (m^2^/g)
Ni	82.6	1.52	2.7	2.7
0.5ReNi	92.5	3.04	5.7	5.3
2ReNi	97.5	2.72	4.3	4.7
0.5RuNi	93.2	2.40	4.4	4.2
2RuNi	98.1	2.23	4.0	3.9

Considering the Re-promoted samples, a small reduction process is evidenced around 360°C that can be attributed to the reduction of rhenium oxides in the catalyst surface (Das et al., 2003; Baranowska et al., 2014; Baranowska and Okal, 2016). The latter peak seems wider in the 2ReNi sample than in the 0.5ReNi sample, which could indicate that rhenium oxides in the 0.5ReNi sample are more isolated, while in the 2ReNi sample are in stronger contact with NiO. The absence of rhenium oxide peaks in the XRD analysis could support this statement.

Last, as it was observed with the RuNi samples that the addition of Re shifts the reduction process of NiO to lower temperatures and increases the reducibility of the sample (Table 3). However, the interaction between Re and Ni seems lower than the Ru–Ni interaction since in both ReNi samples, there is still a wide and intense signal around 600°C which denotes NiO with no interaction with the promoter.

**FIGURE 4:**
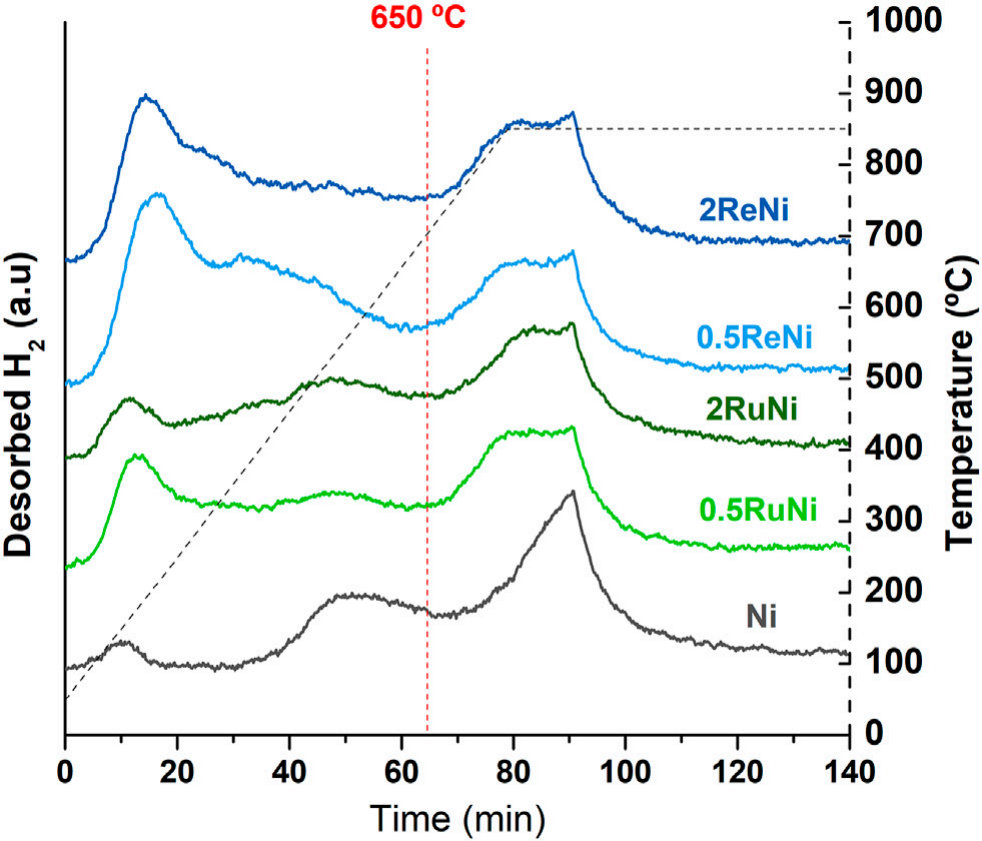
H_2_-TPD profiles of the synthesized samples.

To compare the metallic surface area and dispersion of reduced samples, H_2_-TPD analyses were conducted as shown in Figure 4. H_2_-TPD profiles show three main desorption regions. The first one around 200°C has been described as hydrogen desorbed from metallic particles. The second one around 500°C has been described as hydrogen located in the subsurface layer, and the last one around 800°C corresponds to H_2_ in the support by the spillover effect. In line with the previous information, it is clearly noticed that the amount of available metallic sites is larger in the Re–Ni samples, followed by the Ru–Ni samples and, lastly, the monometallic sample.

As desorption peaks for hydrogen atoms in the metallic surface and subsurface are detected below 650°C (Velu and Gangwal, 2006; Bang et al., 2016; Luisetto et al., 2017), only these peaks were considered for the calculation of hydrogen uptake by the metallic surface (Table 3). Results show that the addition of both promoters increases the chemisorbed H_2_ and, hence, the dispersion and the metallic surface of the catalyst. Regarding the amount of promoter, it seems that 0.5% is better than 2% in order to increase the metallic dispersion. Such findings are in line with what has been reported as the effect of the noble metals as promoters to the Ni catalyst (Daorattanachai et al., 2018).

**FIGURE 5:**
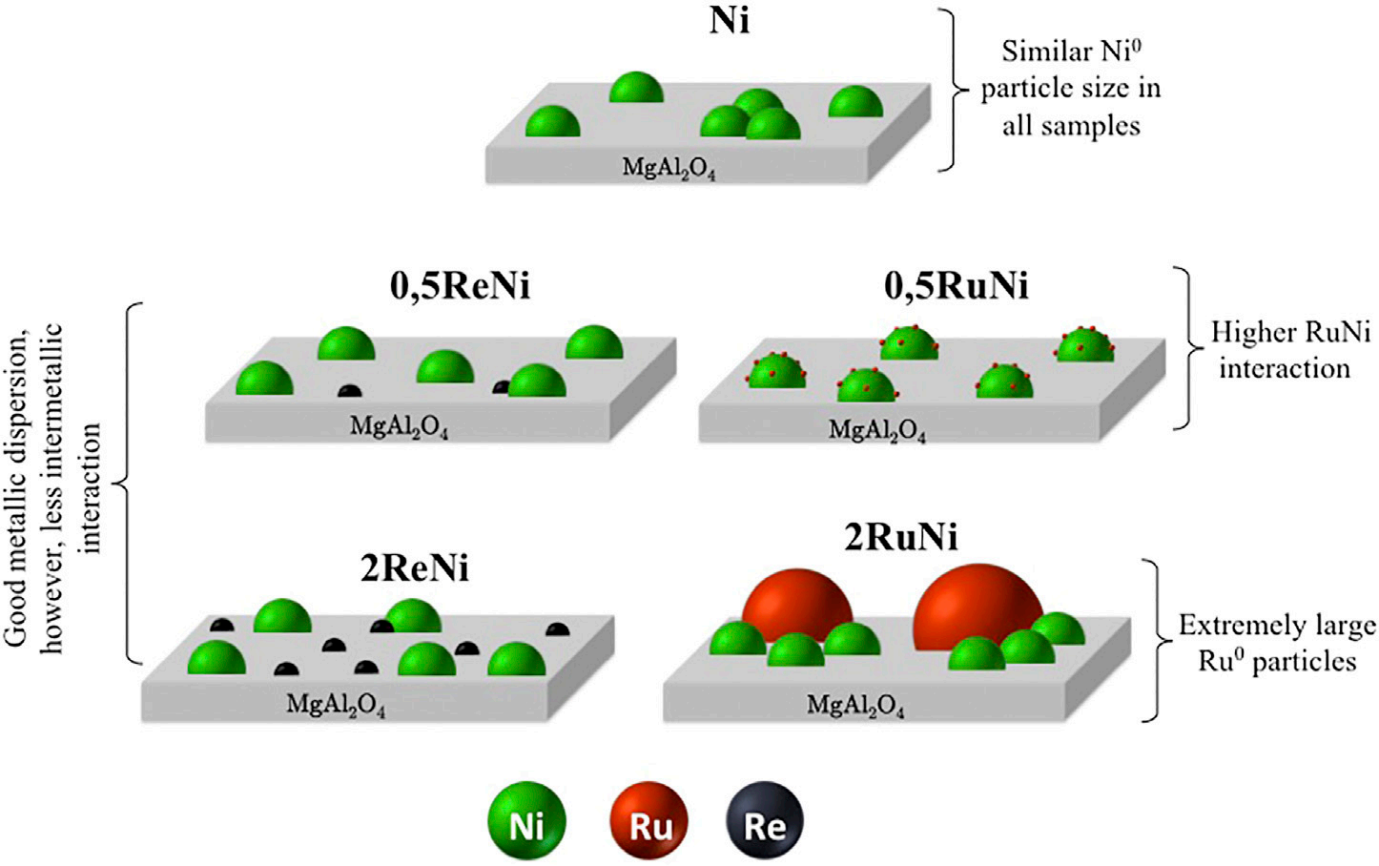
Graphical representation of the synthesized samples.

Figure 5 shows a graphical representation of the synthesized catalyst in order to summarize the principal differences of the samples evidenced by XRD, H_2_-TPR, and H_2_-TPD. As shown, all samples present a very similar Ni^0^ particle size and dispersion; however, crucial differences are spotted in the bimetallic interactions, where the 0.5RuNi sample stands out.

### Catalytic Activity

Table 4 shows the CO_2_ and CH_4_ conversions of the DRM reaction performed at 750°C during 6 h of reaction. The monometallic sample presents a CO_2_ and CH_4_ conversion around 73 and 61%, respectively. These conversion values increase to 81 and 75%, respectively, when the catalyst is doped with 0.5% of Ru, and to 79 and 67%, respectively, when doped with 2% of Ru. The boost in catalytic activity evidenced by the 0.5RuNi sample could be explained by the strong Ni–Ru interaction evidenced in the TPR analysis, by the presence of Ru, which has been described as an active phase on DRM and by the excellent dispersion of both metals, demonstrated by the XRD analysis and H_2_-TPD studies. However, the amount of promoter seems critical since the big particles of RuO_2_ evidenced by XRD and TPR in the 2RuNi sample did not benefit the catalytic activity as much as the well-dispersed and small Ru clusters in the 0.5RuNi catalyst.

**TABLE 4:** Catalytic performance of the synthesized samples.

Sample	Conversion (%)^a^		Syngas H_2_/CO ratio	Selectivity (%)^a^		Deactivation degree (%)^b^	
	CO_2_	CH_4_		H_2_	CO	CO_2_	CH_4_
Ni	73.0 ± 0.4	60.7 ± 0.5	0.8	91.9 ± 0.2	72.2 ± 0.4	2.6	2.6
0.5RuNi	81.8 ± 0.7	75.0 ± 0.9	0.9	100 ± 0.2	89.5 ± 0.6	6.4	5.0
2RuNi	79.1 ± 0.8	67.6 ± 0.9	0.9	100 ± 0.8	85.4 ± 0.9	10.1	7.9
0.5ReNi	77.4 ± 0.3	64.0 ± 0.3	0.8	94.8 ± 0.2	78.6 ± 0.3	8.1	6.5
2ReNi	75.3 ± 1.1	62.1 ± 1.2	0.8	94.2 ± 0.3	76.2 ± 1.2	11.2	8.7

a After 6 h reaction.

b Deactivation degree after 50 h **=** $\frac{{\left[\mathrm{Xi}\right]}_{1h}-{\left[\mathrm{Xi}\right]}_{50h}}{{\left[\mathrm{Xi}\right]}_{1h}}\times 100$, where [X] = conversion *i* = CH_4_ or CO_2_.

Regarding the Re-doped samples, CO_2_ conversions are around 76% and CH_4_ conversions are around 63% with both amounts of promoter. Results are in agreement with the expected outcome since Re has been described mainly as a promoter on the gasification of carbon deposits, but not as an active phase for CH_4_ that can drastically change the catalytic activity.

**FIGURE 6:**
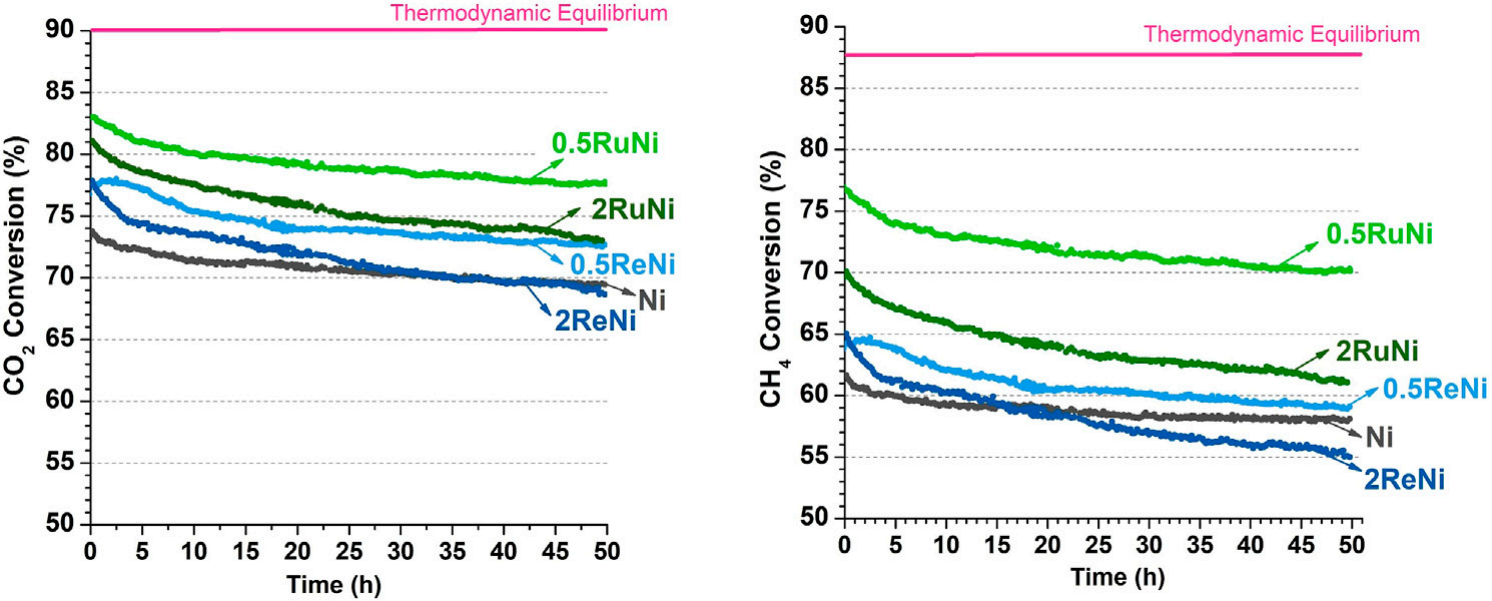
CO_2_ and CH_4_ conversion in a function of time for all catalysts at 750°C and a space velocity of 110 L h^−1^ g^−1^.

Figure 6 shows the CO_2_ and CH_4_ conversion as a function of time for the synthesized samples at DRM during 50 h. The stability test during 50 h allowed the calculation of the deactivation degree of all the synthesized samples (Table 4). The remarkable stability of the monometallic sample stands out, which indicates that the support has a significant effect on the catalyst stability. In this sample, the MgAl_2_O_4_ spinel provides a route for CO_2_ activation, while Ni particles, which are not badly dispersed (Table 3), provide the active sites for CH_4_ activation. This bifunctional mechanism induces the great stability observed in Figure 5.

Regarding the promoted samples, it is observed that promoted catalysts with 0.5% of promoter (0.5ReNi and 0.5RuNi) present the lowest deactivation degree. Re and Ru have been described as active sites for CO_2_ activation (Richardson et al., 2001; Solymosi et al., 2005; Egawa, 2018). Indeed, they are electron-rich species, which can donate electrons to the CO_2_ antibonding orbitals, thus weakening the C–O bond and facilitating the reaction. As an additional benefit of this behavior, the presence of the promoter led to the presence of oxygen atoms that could help to gasify and remove carbon deposits from the CH_4_ decomposition.

There are, however, some differences regarding H_2_ and CO selectivity that could indicate the importance of a secondary process such as the reverse water gas shift reaction. It has been reported that Re changes the acidity of the support in the vicinity of the metals; the slight differences in selectivity, observed in Table 4, could be owed to these changes in acidity (Carvalho et al., 2012; Mohd Arif et al., 2019).

Beyond the comparative trend among the promoted and unpromoted samples, it must be highlighted the fact these experiments are conducted at a remarkably high space velocity. Industrial reformers typically run at much lower space velocities (about an order of magnitude lower). The excellent activity–stability balance achieved under such demanding conditions is highly commendable. Indeed, from the process engineering perspective, running at high space velocity means a significant reduction of the overall reforming reactor volume which would benefit the capital expenditure (CAPEX) in a potential application in an industrial environment. In other words, our multicomponent catalysts are suitable to design compact units for the upgrading of CO_2_/CH_4_ mixtures.

#### Postreacted Sample Analysis

In order to examine the amount of deposited carbon, a TPO analysis was performed after the stability tests (50 h). Figure 6 shows the TPO profiles of the postreacted samples. The majority of the catalysts display a CO_2_ signal from 600°C up to 850°C that could be attributed to the gasification of filamentous carbon with different diameters (McFarlane et al., 2013; Pino et al., 2017). Besides this main peak, the monometallic sample shows a CO_2_ signal around 500°C which could indicate the presence of amorphous carbon (Li and Brown, 2001).

**FIGURE 7:**
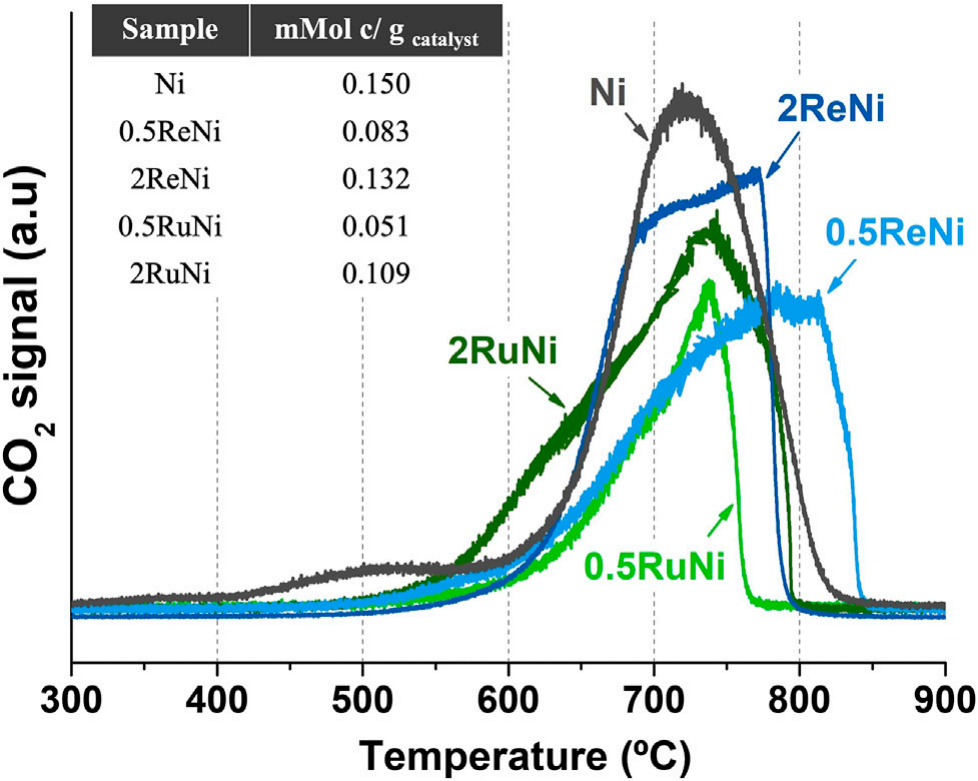
TPO analysis of the postreacted samples.

Re and Ru have been widely reported as good promoters to avoid coke deposition. While some literature indicates that the addition of Re causes an increase in coking initiation temperatures, which decreases carbon deposition (Borowiecki et al., 2008; Mohd Arif et al., 2019), Ru seems to facilitate the gasification of carbon owing to its good activity in CO_2_ dissociation (Egawa, 2018). Results showed that, indeed, Re and Ru could decrease coke deposition, but the amount of promoter is critical. While 0.5% of promoter could reduce in half the deposited coke (Figure 7, Table inset), 2% of promoter does not have this effect. Particle sintering owing to the higher promoter content could explain this result.

## Conclusion

A series of doped Ni catalysts have been successfully applied to the DRM reaction. In general, the presence of a second metal improves Ni reducibility and H_2_ chemisorption, resulting in higher metal dispersion and clear spillover effect. The direct comparison of Ru and Re as promoters for a Ni/MgAl_2_O_4_ catalyst showed that, in both cases, 0.5% of promoter is enough to decrease the carbon deposits in the sample after a 50 h run. However, if boosting the catalytic activity of the catalyst is a priority, only 0.5% of Ru can do the job.

In addition, results opened up a wider panorama regarding very important realistic application. Since our catalysts display excellent catalytic behavior at remarkably high space velocities, it sets the ground for the design of versatile compact CO_2_ conversions units, which are economically more appealing than traditional reforming reactors.

## Data Availability

The raw data supporting the conclusions of this article will be made available by the authors, without undue reservation.
